# Single-Session Therapy by Appointment for the Treatment of Anxiety Disorders in Youth and Adults: A Systematic Review of the Literature

**DOI:** 10.3389/fpsyg.2021.721382

**Published:** 2021-09-01

**Authors:** Vanessa Bertuzzi, Giulia Fratini, Claudia Tarquinio, Flavio Cannistrà, Valentina Granese, Emanuele Maria Giusti, Gianluca Castelnuovo, Giada Pietrabissa

**Affiliations:** ^1^Department of Psychology, Catholic University of Milan, Milan, Italy; ^2^Italian Center for Single Session Therapy, Rome, Italy; ^3^Psychology Research Laboratory, Istituto Auxologico Italiano IRCCS, Milan, Italy

**Keywords:** anxiety disorder, single session therapy, one session, phobia, clinical psychology

## Abstract

**Purpose:** This systematic review provides a summary of the available evidence of the efficacy of single-session therapy (SST) on anxiety disorders in both youth and adults.

**Methods:** PubMed, Scopus, Medline, and Google Scholar databases were search for relevant articles, and the Cochrane Collaboration's tool for assessing the risk of bias in randomized trials was used for transparent reporting of the methodological quality of each selected study.

**Results:** The search of electronic databases identified 18 reports based on rigorous inclusion criteria. Single-session therapy was found superior to no treatment in reducing anxiety symptoms, and similar results were observed while comparing SST to multi-treatment sessions.

**Discussion:** The findings support the benefits of SST in enhancing cognitive, behavioral, and psychological outcomes in both youth and adults suffering from anxiety disorders across treatment conditions and approaches, SST thus appears to be a promising way of providing access to both private and public therapeutic services efficiently and cost-effectively.

**Conclusions:** Single-session therapy is effective in treating anxiety disorders. Further research is required to quantify its cost-effectiveness and deepen the knowledge of effective treatment ingredients for both young people and the adult population suffering from diverse anxiety disorders.

**Systematic Review Registration**: PROSPERO, identifier [CRD42021232024].

## Introduction

It has been increasingly acknowledged not only that anxiety disorders are among the most common mental problems but also that the burden of illness associated with these disorders is substantial across the lifespan—causing a significant global economic burden (Wittchen et al., 2011). Approximately 10–20% of children (median age: 5–10 years) in both the general population and primary care settings report distressing levels of anxiety (Benjamin et al., 2013), and older youth and adults (median age: 24–50 years) account for higher levels of anxiety compared to younger populations thus suggesting that untreated anxiety symptoms get worse over time (Alonso et al., 2018). Indeed, retrospective studies reveal that adults with anxiety disorders report having experienced disturbing anxiety during childhood (Lenze and Wetherell, 2011; Bhatia and Goyal, 2018).

Over the past decades, remarkable advances in the development of effective treatments for anxiety disorders have been made, namely, psychoanalysis, cognitive-behavioral therapies (CBTs), interpersonal psychotherapy, supportive counseling, group therapy, and brief therapy (Hollon and Ponniah, 2010; Pietrabissa et al., 2016; Sanavio, 2017). However, healthcare systems weaknesses—including scarce mental health services and costs of treatment (World Health Organization., 2010)—as well as lack of awareness or stigma perceived by people with anxiety problems, resulting in low use of mental health services for the treatment of mood disorders (Clement et al., 2015). Other challenges involve missed appointments and unexpected dropouts from treatment (Cannistrá et al., 2020). Clinicians and researchers are, therefore, required to look for alternative ways of providing access to both private and public therapeutic services in more efficient and cost-effective ways.

In this regard, the effectiveness of a single-session therapy (SST) has been increasingly investigated and proposed as a potential model to address psychiatric disorders in diverse populations and settings (WHO UN Action et al., 2011). Single-session therapy is an umbrella term which in general indicates a therapeutic intervention conducted by professionals, who use various approaches and techniques to help patients to solve their problems and/or achieve their goal within a single encounter (Paul and van Ommeren, 2013).

Single-session therapy adopts a one-session-at-a-time approach to treatment, and each session of therapy is considered as one self-contained psychotherapy with a beginning, middle, and end (Talmon, 1990; Hoyt and Talmon, 2014; Hoyt et al., 2021). In SST, the therapist adopts a single-session mindset, which involves avoiding assumptions about future meetings with the patient and using his/her resources to move him/her in the direction of his/her goal (Talmon, 1990). This mindset creates the expectation, for both person and therapist, that change is possible in one session.

Existing studies reported improvements of between 71 and 88% in mental health problems following a single therapeutic encounter in both youth and adults (Bloom, 2001; Campbell, 2012), and evidence exists for its specific positive impact on anxiety and panic disorders (Vujanovic et al., 2012; Schleider and Weisz, 2016, 2018) and phobias (Ollendick et al., 2009, 2015; Oar et al., 2015). Such findings support the possibility that SST might be capable of providing significant clinical benefits while meeting the needs of the patients, especially when resources are limited (i.e., public sector). Accordingly, the literature examining the relationship between the number of treatment sessions and rates of change suggest that the greater improvement occurs at the beginning of therapy and decreases over the treatment course (Hansen and Lambert, 2003) and that longer treatments do not always translate to superior clinical outcomes (Weisz et al., 2017).

Single-session therapy is well-supported by decades of research (Silverman and Beech, 1984; Talmon, 1990; Hoyt et al., 1992; Hoyt and Talmon, 2014). A review of studies suggests that the greatest features of SST are its ability to be clinically effective and to be perceived by people as sufficient and helpful (Hymmen et al., 2013). However, the authors made no distinction between planned or walk-in (which do not allow scheduling of appointments and are intended to offer one session with no follow-up with the same therapist) form of SST, neither they selected studies comparing the treatment effects with a control group or focused on a specific psychological outcome (i.e., anxiety).

To provide a more reliable evidence summary over the impact of SST on mood disorders that might help clinicians to offer timely and informed interventions within the health care settings, the present contribution employed a systematic methodology to review the literature on planned single-session interventions for both the youth and adult populations with regard to findings from controlled research studies and efficacy for outcomes across anxiety disorders using narrative and qualitative methods.

## Methods

This systematic review was registered with PROSPERO ID. CRD42021232024. Data extraction, critical appraisal, and qualitative synthesis were in line with established systematic review and qualitative synthesis methods (Khan et al., 2003), and were performed following the Preferred Reporting Items for Systematic Reviews and Meta-Analyses (PRISMA) statement (Page et al., 2021).

### Search Strategy

The PubMed, Scopus, Medline, Google Scholar, and PsychINFO databases were searched between March 12, 2021 and March 14, 2021. Following the PICO elements (Huang et al., 2006), the search strategies combined key terms and Medical Search Headings (MESH) terms for the concepts of: “single session therapy” or “one session,” and “anxiety disorder” or “selective mutism” or phobia or “panic disorder” or “panic attack” or “agoraphobia” or “anxiety” or “worry” or “fear” or “distress.” Boolean and truncation operators were used to combining search terms more systematically and to list documents containing variations on search terms, respectively (Johnson et al., 2002). Search syntax was modified as appropriate for each database.

### Inclusion and Exclusion Criteria

Only original articles that (1) employed a randomized controlled trial (RCT) design, (2) to test the impact of a planned single therapeutic encounter, and (3) in decreasing anxiety symptoms were included. Contributions were excluded if (1) evaluated the impact of walk-in services, (2) anxiety disorders were not primary outcomes, and (3) considered only biomedical outcomes.

No restrictions have been placed for the language of publication, age of the sample, and year of publication.

### Study Selection

Following the search and exclusion of duplicates, two reviewers (authors GF and CT) independently screened the eligibility of the articles first based on the title and the abstract, and the full text according to the inclusion criteria. Authors VB and GP resolved disagreements. Following Smith's recommendation (Smith et al., 2011), the review team included at least one person with methodological expertise in conducting systematic reviews (GP, VB) and at least two experts on the topic under review (FC, GP).

**Figure 1:**
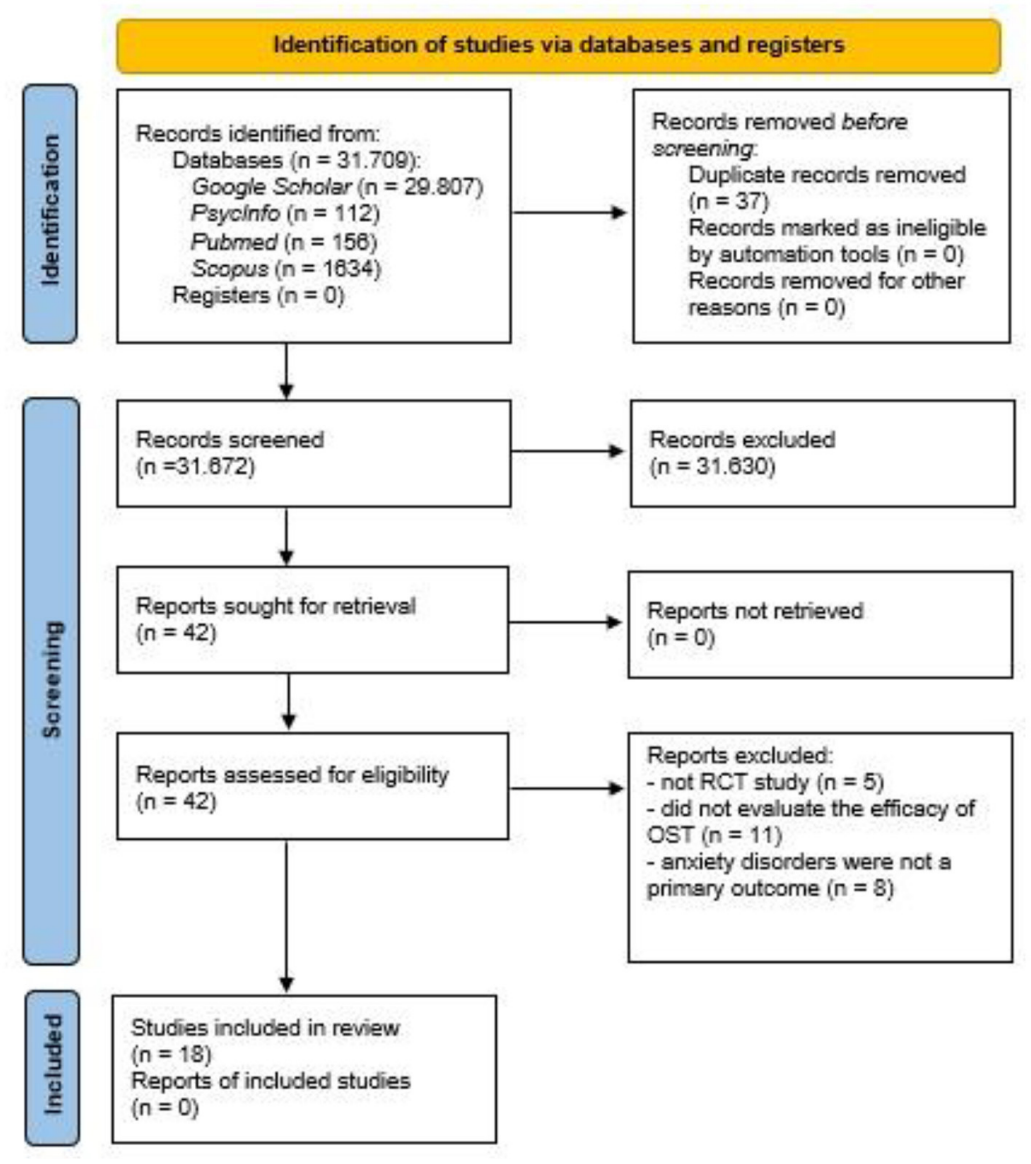
PRISMA flow-chart.

The search of electronic databases identified 31,709 records, of which 37 were duplicates, and 31,630 records were excluded based on information from the title and abstract. The remaining 42 articles were evaluated for inclusion by reviewing their full text and resulted in the exclusion of 24 records for the following reasons: (1) were not RCT studies (*n* = 5) (Öst, 1989; Maltby, 2001; Robbins et al., 2015; Miloff et al., 2019; Wannemueller et al., 2020), (2) did not evaluate the efficacy of SST (*n* = 11) (Öst et al., 1991, 1997b; Beidel et al., 2000; Kim et al., 2002; Masia-Warner et al., 2005; Andersson et al., 2009, 2013; Nilsson et al., 2011; Nielsen et al., 2016; Ryan et al., 2017; Lindner et al., 2019), and (3) anxiety disorders were not primary targeted outcomes (*n* = 8) (Heading et al., 2001; Basoglu et al., 2005; Reinecke et al., 2013; Waters et al., 2014; Goetz and Lee, 2015; Schleider and Weisz, 2016; Schleider et al., 2019; Jiang et al., 2020). Eighteen records ultimately entered the systematic review (Öst et al., 1992, 1997a, 2001a,b; De Jongh et al., 1995; Öst, 1996; Götestam, 2002; Huey and Pan, 2006; Nuthall and Townend, 2006; Haukebo et al., 2008; Ollendick et al., 2009, 2015, 2017; Vika et al., 2009; Muller et al., 2011; Moldovan and David, 2014; Hyett et al., 2018; Hemyari et al., 2019). References for the 18 remaining articles were further screened for relevant records, but none was found. The flowchart presented in Figure 1 provides step-by-step details of the study selection process.

### Assessment of Risk of Bias

The Cochrane Collaboration's tool for assessing the risk of bias in randomized trials (Higgins et al., 2011) was used to assess the methodological quality of each selected study. The tool covers the risk of bias arising from six domains, namely, (1) selection bias (random sequence generation and allocation concealment), (2) performance bias (blinding of participants and personnel), (3) detection bias: (blinding of outcome assessment), (4) attrition bias (incomplete outcome data), (5) reporting bias (selective reporting), and (7) other bias. Within each domain, assessments are made for one or more items, which may cover different aspects of the domain or different outcomes. For each item, the tool involves assigning a judgment of high, low, or unclear risk of material bias.

The assessment was conducted independently by two authors (GF and CT), and any disagreements were resolved by a third author (VB).

### Data Extraction and Synthesis

Two authors (GF and CT) independently extracted the following data from included studies: first author and year of publication, country, the aim of the study, target problem, setting, sample size, age and gender of the participants, study outcomes, and measurements, follow-up time points, main results. The two reviewers discussed any discrepancies, and, if necessary, consulting a third team member (author VB) to reach a final decision (Table 1). Extracted data were collated to produce a qualitative synthesis of the effectiveness of SST for anxiety disorders.

**Table 1:** Characteristics of the included studies.

**Author, Year**	**Country**	**Study aim**	**Target problem (***n***/%)—DSM**	**Intervention (***n***)—duration**	**Comparator (***n***)—duration**	**Sample size (***n***)**	**Women gender:** ***n*** **(%)**	**Age:Mean (SD); Range**
**Youth**								
Ollendick et al., 2009	USA/SE	To compare CBT exposure with EST and a WL control for specific phobias	Specific phobias | Animal phobia: dogs (49), spiders (15), wasps (9), birds (5), snakes (4), ants (4), insects (4), spiders (11), bees (4), cats (1), snails (1), and other animals (2); Miscellaneous phobias: dark or being alone (20), heights (3), elevators or enclosed spaces (16), loud noises (8), thunderstorms (22), costumed characters (2), flying (3), water (3), mushrooms (1), and rain (1), costumed characters (2), vomiting or choking (2), taxidermy-prepared animals (2); and other phobias (2)—**DSM-IV**	CBT exposure (85)**—**3 h	EST (70)**—**3 h vs. WL (41)	196	107 (54.6%)	11.0 (0); 7–16
Ollendick et al., 2015	USA	To compare two forms of CBT exposure (youth alone vs. youth with a parent) for specific phobias	Specific phobias | Animal phobia (30); Environmental phobia (51); Situational phobia (2); other phobias (10)—**DSM-IV-TR**	A-OST (51)**—**3 h	CBT exposure (46)**—**3 h	97	50 (51.5%)	8.86 (1.84); 6–15
Ollendick et al., 2017	USA/SE	To compare CBT exposure with EST for specific phobias	Specific phobias | Animal phobia (101); Environmental phobia (38); Situational phobia (2)—**DSM-IV**	CBT exposure (86)**—**3 h	EST (79)**—**3 h	165	61 (37%)	10.37 (2.12); 7–16
Öst et al., 2001b	SE	To compare two forms of CBT exposure (youth alone vs. youth with a parent) with a WL control for specific phobias	Specific phobias | Animal phobia dogs (10), spiders (9), snakes (5), ants (3), birds (1), snails (1), and insects (1); Miscellaneous phobias: injections (12), enclosed spaces (10), blood (2), thunderstorms (2), deep water (1), loud noise (1), mummies (1), and yogurt (1)—**DSM-IV**	CBT exposure: child alone (21) vs. child with a parent (20)**—**3 h	WL (19)	60	37 (61%)	11.7 (2.8); 7–17
**Adults**								
De Jongh et al., 1995	NL	To compare CRI with II and WL for dental phobia	Dental phobia—**DSM-IV**	CRI (15)**—**1 h	II (14) −1 h vs. WL (23)	52	27 (51.92%)	18–65
Götestam, 2002	NO	To compare a modeled version of CBT exposure with direct CBT-exposure and video CBT exposure for spider phobia	Spider phobia (38)—**DSM-III-R**	CBT *model* exposure (13)**—**2 h	CBT exposure (14) vs. CBT *video* exposure (11)**—**2 h	38	35 (92%)	30.3; 19–52
Haukebo et al., 2008	NO	To compare OST-CBT exposure with a five-session treatment and WL for dental phobia	Dental phobia—**DSM-IV**	CBT exposure (10)**—**NR	Multi-session (*n* = 5) CBT Exposure (10) vs. WL (20)**—**NR	40	26 (65%)	34.9 (10.5); 19–60
**Adults**								
Hemyari et al., 2019	IRA	To compare OST-CBT exposure with multiple-session therapy for rat phobia	Rat phobia—**DSM 5**	CBT exposure (20)**—**3 h	Multi-session (*n* = 4) CBT exposure (20)**—**8 h	40	40 (100%)	20.97 (1.25); 18–24
Huey and Pan, 2006	USA	To compare a culturally adapted CTB exposure with standard treatment and manualized self-help for animal phobias	Spiders (80%), crickets, worms, or dead fish (20%)—**DSM–IV**	OST-CA (5)**—**3 h	OST-S (4) vs. manualized self-help (6)**—**3 h	15	10 (67%)	25.5
Hyett et al., 2018	AU	To compare group-based IR with VR and WL for social anxiety	Social anxiety—**DSM-IV**	IR (17)**—**90 min	VR (22)**—**90 min vs. WL (19)	58	39 (67%)	35.22 (14.98)
Moldovan and David, 2014	RO	To compare VR-CBT with WL for social phobia, flight phobia, and acrophobia	Social phobia, flight phobia, and acrophobia—**DSM-IV**	VRCBT (16)**—**4/5 h	WL (16)**—**4/5 h	32	15 (46.8%)	>18
Muller et al., 2011	CH	To compare computer-based self-help CBT treatments for spider phobia	Spider phobia—**DSM IV**	CBE**—**spider pictures (18)**—**27 min	CBE**—**neutral pictures (18)**—**27 min	36	36 (100%)	23.17 (4.21); 18–34
Nuthall and Townend, 2006	UK	To compare CBT with WL for panic disorder	Panic disorder—**DSM IV**	CBT (12)**—**45 min	WL (9)**—**NR	36	17 (63%)	36.80; 18–58
Öst et al., 1992	SE	To compare OST-CBT exposure with a five-session treatment for specific phobias	Simple phobia and injection phobia—**DSM-III and DSM-III-R**	CBT exposure (20)**—**3 h	Multi-session (*n* = 5) CBT exposure (20)**—**5 h	54	35 (64.8%)	26.90 (8.30); 18–51
Öst, 1996	SE	To compare small to large group-based OST-CBT exposure and modeling for spider phobia	Spider phobia—**DSM-III-R**	CBT exposure and modeling | small group**—**3 h	Exposure and modeling | large group**—**3 h	42	42 (100%)	32.50 (8.80); 18–55
Öst et al., 1997a,b	SE	To compare OST-CBT exposure and cognitive restructuring with a five-session treatment for flying phobia	Flying phobia—**DSM-IV**	CBT exposure and cognitive restructuring (14)**—**3 h	Multi-session (*n* = 5) CBT exposure and cognitive restructuring (14)**—**6 h	28	NR	39.0 (9.50); 22–60
Öst et al., 2001a	SE	To compare OST-CBT exposure with five-sessions CBT-exposure treatment, five-sessions CBT treatment, and WL for claustrophobia	Claustrophobia—**DSM-IV**	CBT exposure (10)**—**3 h	Multi-session (*n* = 5) CBT exposure (11) vs. multi-session (5) CBT (11)**—**5 h vs. WL (18)	46	NR	41.30 (9.60); 22–60
Vika et al., 2009	SE	To compare OST-CBT exposure with a five-session treatment for intra-oral injection phobia	Intra-oral injection phobia—**DSM-IV**	CBT exposure (28)**—**NR	Multi-session (*n* = 5) CBT exposure (27)**—**NR	55	NR	32.5 (12.2); 18–62
**Author, Year**	**Follow-up points**	**Primary outcomes**	Primary outcomes_measures^*^	**Primary outcomes_results^†^**	**Secondary outcomes**	**Secondary outcomes_measures**		**Secondary outcomes_results^†^**
**Youth**								
Ollendick et al., 2009	T1 = pre-treatment; T2 = 1-week; T3 = 6-month (WL re-randomized to OST or EST groups)	Anxiety level; behavioral response; distress; treatment satisfaction	CSR of ADIS-C/P; BAT; SUDS; TSS	CSRs: significant within-gr decrease was observed from T1 to T2 (*p* < 0.001) and from T2 to T3 (*p* < 0.001) across groups. Significant between-gr difference was observed from T1 to T2 (*p* < 0.0001) and from T2 to T3 (*p* < 0.01) in favor of OST. (OST_T1meanCRS = 6.04 ± 1.01 vs. EST_T1meanCRS = 5.90 ± 0.92 vs. WL_T1meanCRS = 5.98 ± 1.01; OST_T2meanCRS =3.47 ± 1.77 vs. EST_T2meanCRS = 4.83 ± 1.52 vs. WL_T2meanCRS = 5.98 ± 1.01; OST_T3meanCRS = 3.19 ± 1.94 vs. EST_T3meanCRS = 4.07 ± 1.89). Diagnosis free: significant between-gr difference was observed from T1 to T2 (*p* < 0.001) [OST: 55% (*n* = 47) vs. EST: 23%; (*n* = 16) vs. WL: 2%; (*n* = 1)], at T2 (*p* < 0.001) [OST: 52% (*n* = 53) vs. EST: 21%; (*n* = 18)], and at T3 (*p* < 0.05) [OST: 49% (*n* = 50) vs. EST: 35%; (*n* = 30)]. BAT: significant within-gr decrease was observed from T1 to T2 (*p* < 0.001), and from T2 to T3 (*p* < 0.001). SUDS: significant between-gr (*p* < 0.05), and between-country (*p* < 0.01) differences were observed from T1 to T2 in favor of OST (vs. EST) and the US (vs. Sweden) sample, respectively.	Fear; General anxiety; depression; Parents' perceptions of their children's problems	FSSC-R; MASC; GDI; CBCL		*CBCL:* Significant between-gr difference was observed in favor of OST and the US samples at T3 (*p* < 0.01). Significant within-group decreases were observed over time for the CBCL-anxious/depressed scale, CBCL-internalizing scale, MASC, and FSSC-R.
**Youth**								
Ollendick et al., 2015	T1 = pre-treatment; T2 = 1-week; T3 = 1-month; T4 = 6-month	Anxiety level; self-efficacy; treatment satisfaction	CSR of ADIS–C/P; PCIR and CCIR; CIR; PSE; PTS; and CTS	CSRs: significant within-gr decrease was observed from T1 to T2 (*p* < 0.001), from T2 to T3 (*p* < 0.001), and from T3 to T4 (*p* = 0.04). PCIR and CCIR: Significant within-gr decreases were observed over time (*p* < 0.001). Diagnosis free: no significant between-gr difference was observed, but a trend (*p* = 0.07) at T4 in favor of OST (67.39%) vs. A-OST (49.02%). PSE: significant between-gr difference was observed from T1 to T2 (*p* < 0.001), from T2 to T3 (*p* < 0.001), and from T3 to T4 (*p* = 0.011) in favor of OST. (OST_T1meanPSE = 11.12 ± 0.40 vs. A-OST_T1meanPSE = 13.17 ± 0.30; OST_T2meanPSE = 10.73 ± 0.44 vs. A-OST_T2meanPSE = 13.46 ± 0.42; OST_T3mean PSE = 11.33 ± 0.51 vs. A-OST_T3meanPSE = 13.13 ± 0.48) Older age significantly predicted CSR (*p* = 0.02) and PCIR across time (*p* = 0.05).	NR	NR		NR
Ollendick et al., 2017	T1 = pre-treatment; T2 = 1-week; T3 = 6-month	Anxiety level; phobic beliefs	CSR of ADIS-C/P; PBS	Significant within-gr decrease in PBS was found at T2 in favor of OST (*p* < 0.001). Greater change in the PBS predicted lower CSRs at T2 and T3 (*p* < 0.001).	NR	NR		NR
**Youth**								
Öst et al., 2001b	T1 = 1-week pre-treatment; T2 = 1-week; T3 = 12-month	Anxiety level; behavioral response; fear; general anxiety; state and trait anxiety; fear of anxiety symptoms; depression	CSR of ADIS-C/P; BAT; FSSC-R; RCMAS; STAIC; CASI; GDI | **BP; HR**	Significant between-gr difference was found from T1 to T2 (*p* < 0.001) for BAT**—**maintained over time. The OST gr. did better than the A-OST gr. (*p* < 0.02) and the WL gr. (*p* < 0.0001); and the A-OST did better than the WL (*p* < 0.0001). Self-rating of anxiety: both active treatments showed significant improvement (vs. WL) that was maintained at T3. Significant within-gr decreases were observed for the FSSC-R, CASI, and STAIC-Trait from T1 to T2 (*p* < 0.001).	NR	NR		NR
**Adults**								
De Jongh et al., 1995	T1 = 1-month pre-treatment; T2 = 1-week pre-treatment; T3 = Post-treatment; T4 = 1-month; T5 =12-month	Dental anxiety; dental cognition; behavioral coping style	DAS; DCQ; MBSS	DCQ: at T3, significant within-gr differences (*p* < 0.001) were found in all conditions, with better results in CRI. At T4, significant between-gr differences were observed in favor of CRI (*p* < 0.005). DAS: at T3, significant within-gr differences (*p* < 0.001) were observed with better results for CRI. At T4, within-gr differences (*p* < 0.001) were found in favor of CRI. At T5, CRI showed greater improvement compared to the II and WL conditions. CRImeansMBSS-listen the tape: after 3.3 ± 2.8 days vs. IImeansMBSS-listen the tape: after 5.6 ± 3.0 days; *p* < 0.05; CRImeansMBSS-time for listening: 10.2 ± 12.2 min vs. IImeansMBSS-time for listening: 2.9 ± 5.8 min (*p* = 0.06) CRImeansMBSS-listened on more occasions: 2.7 ± 1.9 times vs. IImeansMBSS-listened on more occasions: 0.9 ± 1.2 times (*p* < 0.01).	NR	NR		NR
**Adults**								
Götestam, 2002	T1 = pre-treatment; T2 = 1-week; T3 = 6-month; T4 = 12-month	Phobic cognitions; Somatic reactions; Self-efficacy; Evaluation of treatment	CQ; BSQ; *ad hoc* SET; *ad hoc* QETR	CQ: significant within-gr differences (*p* = 0.001) were found in all conditions at T2, with better maintenance at T3 and T4 for the direct exposure condition. BSQ: significant within-gr differences (*p* = 0.001) were found in all conditions at T2, with a better tendency for direct exposure condition. SET: significant within-gr differences (*p* = 0.001) were found in all conditions at T2, with best results for direct exposure condition.	NR	NR		NR
Haukebo et al., 2008	T1 = pre-treatment; T2 = post-treatment (After 5 weeks WL participants were randomly assigned to OST-CBT-E or 5-CBT-E and post-treatment assessment occurred 1 week after treatment); T3 = 12-month	Dental anxiety; dental fear; dental beliefs; maximum anxiety; positive and negative thoughts. behavioral response	DAS; DFS; DBS-R; MA; PT; NT; BAT	T1–T2 significant between-gr (*p* < 0.01) and within-gr differences (*p* < 0.0001) were found for DAS, DFS, and DBS-R in favor of both treatment gr compared to WL Significant between-gr (*p* < 0.05) and within-gr differences (*p* < 0.0001) were found for PT, MA, and NT (*p* < 0.0001) were found in favor of both treatment gr compared to WL. Significant between-gr and within-gr differences (both *p* < 0.0001) were found for BAT in favor of both treatment gr. compared to WL T2–T3 (after WL randomization) significant between-gr (*p* < 0.05) were found in favor of 5-CBT-E. Within-gr differences were found in DFS, DAS, DBS-R, PT, NT, and MA (*p* < 0.0001) with similar trajectories in both gr.	NR	NR		NR
**Adults**								
Hemyari et al., 2019	T1 = pre-treatment; T2 = post-treatment	Rat phobia; anxiety disorder; anxiety state; anxiety traits	FRQ; STAI-S; DPSS-R	T1–T2: all measures improved with similar trajectories across gr.—but no significant between-gr and within-gr differences were found. OST-CBT-E_T1meansFRQ = 71.6 ± 23.7 vs. 4-CBT-E_T1measnFRQ = 86.7 ± 12.4; OST-CBT-E_T2meansFRQ = 18.7 ± 20.4 vs. 5-CBT-E_T2measnFRQ = 17.6 ± 12.1; OST-CBT-E_T1meansSTAI-S = 54.0 ± 25.0 vs. 4-CBT-E_T1measnSTAI-S = 57.8 ± 7.2; OST-CBT-E_T2meansSTAI-S = 18.7 ± 20.4 vs. 5-CBT-E_T2measnSTAI-S = 17.6 ± 12.1; OST-CBT-E_T1meansDPSS-R = 45.4 ± 8.6 vs. 4-CBT-E_T1measnDPSS-R = 51.3 ± 7.5; OST-CBT-E_T2meansDPSS-R = 36.4 ± 8.5 vs. 5-CBT-E_T2measnDPSS-R = 41.1 ± 8.8.	NR	NR		NR
Huey and Pan, 2006	T1 = pre-treatment; T2 = 1-week	Phobic anxiety and avoidance; behavioral response; general phobic tendencies; catastrophic thinking; distress	ADIS-IV; BAT; FSS-III; FTQ; SL-ASIA; SUD	T1–T2: significant between-gr differences in favor of OST-CA and OST-S were found for ADIS-IV (*p* < 0.01), BAT (*p* < 0.001), clinician severity (*p* < 0.001), and FTQ (*p* < 0.05) compared to WL. Marginal between-gr differences in favor of OST-CA and OST-S were found for FSS-III (*p* < 0.10) and SUD (*p* < 0.10) compared to WL.	NR	NR		NR
Hyett et al., 2018	T1 = pre-treatment; T2 = post-treatment; T3 = 2-week	Social anxiety; cognitive avoidance; negative self-portrayal; repetitive thinking; stress	SIPS; CAQ; NSPS; RTQ; TSST	IR and VR reported lower levels of SIPS, CAQ, and RTQ, but not statistically significant between-gr and within-gr differences were found at T2 and T3. Statistically significant between-groups differences were reported for NSPS at T2 (*p* = 0.001). TSST improved only in the intervention groups	NR	NR		NR
**Adults**								
Moldovan and David, 2014	T1 = pre-treatment; T2 = post-treatment	Social anxiety; flight anxiety; state and trait anxiety; distress; self-statement during public speaking; fear of negative evaluations; attitudes and beliefs; expectations; acrophobia anxiety; therapeutic alliance	LSAS; FAS; STAI-S; STAI-T; SUD; SSPS-P; SSPS-N; BFNE; FAM-S; FAM-C; ABS-II-R; ABS-II-IR; VAS; AQ; WAI; ITQ; PQ	Significant within-gr differences were found for SUD, SSPS-P, SSPS-N, BFNE, LSAS, FAS, FAM-S, FAM-C (all with *p* < 0.05) in favor of VRCBT condition.	NR	NR		NR
Nuthall and Townend, 2006	T1 = pre-treatment; T2 = post-treatment; T3 = 1-month	Spider fear; behavioral response	FSQ; BAT	T1–T2: significant between-gr difference in favor of CBE-group was found for FSQ (*p* = 0.004) and BAT (*p* = 0.001). T2–T3: CBE-group improvements in FSQ remained stable. CBE_T1meansFSQ = 60.06 ± 14.97 vs. CG_T1meansFSQ = 58.94 ± 21.29; CBE_T2meansFSQ = 44.44 ± 20.85 vs. CG_T2meansFSQ = 52.44 ± 18.32; CBE_T3meansFSQ = 42.24 ± 21.31 vs. CGmeansT3=61.53 ± 18.67. CBE_T1meansBAT = 6.44 ± 1.15 vs. CG_T1meansBAT = 6.78 ± 1.21; CBE_T2meanBAT = 7.39 ± 0.61 vs. CG_T2meanBAT= 6.83 ± 1.34.	NR	NR		NR
Nuthall and Townend, 2006	T1 = pre-treatment; T2 = 1-month; T3 = 3-month	Panic disorder	PDSS-SR	PDSS-SR (total score): Significant within-gr differences were found from T1 and T2 (*p* = 0.004) and from T1 and T3 (*p* = 0.01) in the CBT condition, and from T1 to T2 (*p* = 0.026) in the WL condition.	NR	NR		NR
Öst et al., 1992	T1 = pre-treatment; T2 = post-treatment; T3 = 12-month	Blood phobia; injection phobia (anxiety, avoidance); general phobic tendencies; anxiety, depression; behavioral response	MQ; IPS (anxiety and avoidance); FSS-III BAI; BDI; BAT | **HR; SBP; DBP**	T1–T3: significant within-gr differences were found for IPS, FSS-III, MQ, and BAT (*p* = 0.0001), BAI, BDI, and all physiological measures (*p* < 0.01) with similar trajectories between IGs.	NR	NR		NR
**Adults**								
Öst, 1996	T1 = pre-treatment; T2 = post-treatment; T3 = 12-month	Spider phobia; fear; avoidance; degree of handicap; general phobic tendencies; anxiety; depression; behavioral response	SPQ; SQ; SAAV; SAFEAR; SAHAND; FSS-III; STAI; BDI; BAT | | **HR; SBP; DBP**	T1–T3: significant within-gr differences in physiological measures were found (*p* < 0.0001) in favor of the small group. T2–T3_STAI was lower in the small group compared to the large one. Significant within-gr differences were found in all measures from T2 to T3 (*p* < 0.0001) in favor to the small group.	NR	NR		NR
Öst et al., 1997a,b	T1 = pre-treatment; T2 = 1-week; T3 = 12-month	Flying fear; general phobic tendency; behavioral response; anxiety, depression	FFI; FFS-III; BAT; STAI; BAI; BDI	T1–T3: Significant within-gr differences were found for FFI and FFS-III (*p* < 0.0001), BAI (*p* < 0.05), BDI and STAI-T (*p* < 0.005) BAT: Significant within-gr differences in both groups were found at T2 (*p* < 0.0001); at T3 significant within-gr differences in OST-CBT-E (*p* < 0.05) and marginally in 5-CBT-E were found.	NR	NR		NR
Öst et al., 2001a	T1 = pre-treatment; T2= 1-week; T3 = 12-month	Claustrophobia; general phobic tendencies; behavioral response; anxiety sensitivity; body sensations; agoraphobia cognitions; depression; anxiety	CS; CLQ; FSS-III; BAT; ASI; BSQ; ACQ; BDI; BAI | **HR; SBP; DBP**	T1–T3: Significant within-gr differences were found for CS, CLQ, FSS-III, ACQ, BSQ, BAI, and BDI in favor of the IGs. (*p* < 0.0001). T1–T3_BAT (elevator test): the IGs showed significant within-gr differences compared to WL (*p* < 0.05). T1–T3_BAT (small room test): the IGs showed significant within-gr differences compared to WL (*p* < 0.001), with the OST-CBT-E resulting the more effective IG. T1–T3_BAT (gas mask test) = the IGs showed significant within-gr differences compared to WL (*p* < 0.05), with no differences between IGs. T1–T3: Significant within-group differences were found for SBP (*p* < 0.01) and DBP (*p* < 0.001) in all three BAT situations. Significant within-group differences were found for HR (*p* < 0.0001) in the BAT-elevator and the small room test, but not in the gas mask test.	NR	NR		NR
**Adults**								
Vika et al., 2009	T1 = pre-treatment; T2 = post-treatment; T3 = 12-month	Dental anxiety; behavioral response; blood-injury fear; injection phobia scale-anxiety	DAS; BAT; MQ; IPS-A	T1–T3-DAS: Significant between-gr (*p* < 0.001) and within-gr differences (*p* < 0.0001) were found in favor of 5-CBT-E. T1–T3-IPSA, MQ, BAT: Significant within-gr differences were found in both conditions (with better scores in 5-CBT-E) (all with *p* < 0.0001).	NR	NR		NR

*5-CBT,Five-sessions cognitive behavioral therapy only; 5-CBT-E, five-sessions cognitive behavioral therapy-exposition; A-OST, parent-augmented one-session treatment; ABS-II (R and IR), attitudes and beliefs scale II (rationally and irrationally); ACQ, agoraphobic cognitions questionnaire; ADIS-C/P, anxiety disorders interview schedule for child and parent; AQ, acrophobia questionnaire; ASI, anxiety sensitivity index; AU, Australia; BAI, Beck anxiety inventory; BAT, behavioral approach test; BDI, Beck depression inventory; BFNE, fear of negative evaluation scale; BSQ, body sensations questionnaire; CAQ, cognitive avoidance questionnaire; CASI, children's anxiety sensitivity index; CBCL, child behavior checklist; CBE, computer based exposure treatment; CBT, cognitive behavior therapy; CCIR, clinician improvement rating form; CDI, children's depression inventory; CG, control group; CGAS, children's global assessment scale; CH, Switzerland; CIR, child improvement rating; CLQ, claustrophobia questionnaire; CQ, cognitions questionnaire; CRI, cognitive restructuring intervention; CS, claustrophobia scale; CSR, clinician severity rating; DAS, dental anxiety scale; DASS, depression anxiety stress scales; DBP, diastolic blood pressure; DBS-R, dental belief survey-revised; DCQ, dental cognition questionnaire; DFS, dental fear survey; DPSS-R, disgust propensity and sensitivity scale-revised; EST, educational support therapy; FAM (S and C), flight anxiety modality questionnaire (somatic and cognitive); FAS, flight anxiety situation questionnaire; FFI, fear of flying inventory; FFS, fear of flying scale; FRQ, fear of rats questionnaire; FSQ, fear of spiders questionnaire; FSS-III, fear survey schedule III; FSSC-R, fear survey schedule for children-revised; FTQ, fearful thoughts questionnaire; HADS, hospital anxiety and depression scale; HR, heart rate; IG, intervention group; II, information intervention; IPS, injection phobia scale; IPS-A, injection phobia scale-anxiety; IPTQ, implicit personality theory questionnaire; IR, imagery rescripting; IRA, Iran; ITQ, immersive tendencies questionnaire; LSAS, Liebowitz social anxiety scale; MA, maximum anxiety; MASC, multidimensional anxiety scale for children; MBSS, Miller behavioral style scale; MI, mobility inventory; MQ, mutilation questionnaire; MT; motivation for treatment; NL, Netherlands; NO, Norway; NSB: exposure with no safety behaviors; NSPS, negative self-portrayal scale; NT, negative thoughts; OST, one session treatment; OST-CA, culturally adapted one-session treatment; OST-CBT-E, one session treatment cognitive behavioral therapy-exposition; OST-S, standard one session treatment; PBS, phobic beliefs scale; PCIR, parent clinician improvement rating form; PCSC, perceived control scale for children; PDSS, panic disorder severity scale; PDSS-SR, panic disorder severity scale-self report; PIR, parent improvement rating; PQ, presence questionnaire; PSE, parent self-efficacy; PT, positive thoughts; QETR, questionnaire for evaluation of the treatment results; RCMAS, revised children's manifest anxiety scale; RO, Romania; RTQ, repetitive thinking questionnaire; SAAV, self-assessment of avoidance; SAFEAR, self-assessment of fear; SAHAND, rating of overall degree of handicap; SBP, systolic blood pressure; SCSC, secondary control scale for children; SE, Sweden; SET, self-efficacy tasks; SIPS, social interaction phobia scale; SL-ASIA, Suinn-Lew Asian self-identity acculturation scale; SMFQ-C, short mood and feelings questionnaire child version; SMFQ-P, short mood and feelings questionnaire parent version; SPQ, spider fear questionnaire; SQ, spider questionnaire; SSPS-N, negative self-statements during public speaking scale; SSPS-P, Positive self-statements during public speaking scale; STAI-Y, state-trait anxiety inventory-Y form; STAIC, state-trait anxiety inventory for children; SUD, subjective units of distress; TSS, treatment satisfaction survey; TSST, trier social stress test; UK, United Kingdom; USA, United States; VAS, visual analogue scale; VR, verbal restructuring; VRCBT, virtual reality cognitive behavioral therapy; WAI: working alliance inventory; WL, waiting list*.

* *Psychological data measurements in bold. Where not otherwise specified, times are expressed in months*.

† *Only significant p-values were reported. NR, not reported*.

## Results

### The Methodological Quality of the Included Studies: the Cochrane Collaboration's Tool

Eight out of the 18 selected articles (Öst et al., 1992; De Jongh et al., 1995; Haukebo et al., 2008; Ollendick et al., 2009, 2015, 2017; Muller et al., 2011; Hyett et al., 2018) displayed an unclear methodologically quality (with low or unclear risk of bias for all domains), while the other 10 records (Öst, 1996; Öst et al., 1997a, 2001a,b; Götestam, 2002; Huey and Pan, 2006; Nuthall and Townend, 2006; Vika et al., 2009; Moldovan and David, 2014; Hemyari et al., 2019) had a weak methodological quality (with high risk of bias for one or more key domains). No article presented a strong methodologically quality. Table 2 shows the rating of each selected study.

**Table 2:** Quality assessing rating with Cochrane collaboration's tool.

**Author, Year**	**D1**	**D2**	**D3**	**D4**	**D5**	**D6**	**D7**	**TOTAL**
**Studies on Youth**								
Ollendick et al., 2009	Low risk	Low risk	Low risk	Low risk	Low risk	Low risk	Unclear risk	UNCLEAR
Ollendick et al., 2015	Unclear risk	Low risk	Low risk	Low risk	Low risk	Low risk	Low risk	UNCLEAR
Ollendick et al., 2017	Unclear risk	Unclear risk	Unclear risk	Low risk	Low risk	Low risk	Low risk	UNCLEAR
Öst et al., 2001b	Unclear risk	High risk	Unclear risk	Low risk	Unclear risk	Low risk	Low risk	HIGH
**Studies on Adults**								
De Jongh et al., 1995	Unclear risk	Low risk	Unclear risk	Low risk	Low risk	Low risk	Low risk	UNCLEAR
Götestam, 2002	Low risk	High risk	Unclear risk	Low risk	High risk	Low risk	High risk	HIGH
Haukebo et al., 2008	Unclear risk	Unclear risk	Low risk	Low risk	Low risk	Low risk	Low risk	UNCLEAR
Hemyari et al., 2019	Unclear risk	High risk	High risk	Low risk	Low risk	Low risk	High risk	HIGH
Huey and Pan, 2006	Unclear risk	Unclear risk	Unclear risk	Low risk	High risk	Low risk	Unclear risk	HIGH
Hyett et al., 2018	Low risk	Low risk	Unclear risk	Low risk	Unclear risk	Low risk	Low risk	UNCLEAR
Moldovan and David, 2014	Low risk	Low risk	Unclear risk	Low risk	Low risk	Low risk	High risk	HIGH
Muller et al., 2011	Unclear risk	Unclear risk	Unclear risk	Low risk	Unclear risk	Low risk	Low risk	UNCLEAR
Nuthall and Townend, 2006	High risk	Unclear risk	Unclear risk	Low risk	Low risk	Low risk	Unclear risk	HIGH
Öst et al., 1992	Unclear risk	Unclear risk	Unclear risk	Low risk	Low risk	Low risk	Unclear risk	UNCLEAR
Öst, 1996	Unclear risk	Unclear risk	High risk	Low risk	Low risk	Low risk	High risk	HIGH
Öst et al., 1997a	Unclear risk	Unclear risk	Unclear risk	Low risk	Low risk	Low risk	High risk	HIGH
Öst et al., 2001a	Unclear risk	Unclear risk	High risk	Low risk	Low risk	Low risk	High risk	HIGH
Vika et al., 2009	Low risk	Unclear risk	Low risk	Low risk	Low risk	Low risk	High risk	HIGH

*D1, selection bias: random sequence generation; D2, selection bias: allocation concealment; D3, performance bias: blinding of participants and personnel; D4, detection bias: blinding of outcome assessment; D5, attrition bias: incomplete outcome data; D6, reporting bias: selective reporting; D7, other bias: anything else, ideally prespecified*.

### Characteristics of the Included Studies

Selected studies were published between 1992 (Öst et al., 1992) and 2019 (Hemyari et al., 2019). Most of the investigations (*n* = 6) were conducted in Sweden (Öst et al., 1992, 1997a, 2001a,b; Öst, 1996; Vika et al., 2009), two studies were conducted both in Sweden and in the USA (Ollendick et al., 2009, 2017) while two studies in the USA only (Huey and Pan, 2006; Ollendick et al., 2015). The other selected studies were conducted in Norway (*n* = 2) (Götestam, 2002; Haukebo et al., 2008), The Netherlands (*n* = 1) (De Jongh et al., 1995), Iran (*n* = 1) (Hemyari et al., 2019), Australia (*n* = 1) (Hyett et al., 2018), Romania (*n* = 1) (Moldovan and David, 2014), Switzerland (*n* = 1) (Muller et al., 2011), and the UK (*n* = 1) (Nuthall and Townend, 2006). The sample size varied from a minimum of 15 subjects (Huey and Pan, 2006) to a maximum of 196 (Ollendick et al., 2009) participants across studies. The mean age range of the individuals is from 10.37 (SD = 2.12) (Ollendick et al., 2017) to 41.30 (SD = 9.60) years (Öst et al., 2001a). Most of the investigations included participants of both genders, except for six studies that did not specify the gender of the sample (*n* = 3) (Öst et al., 1997a, 2001a; Vika et al., 2009) or included only women (*n* = 3) (Öst, 1996; Muller et al., 2011; Hemyari et al., 2019).

Four studies investigated the impact of SST for anxiety disorders among youth (Öst et al., 2001b; Ollendick et al., 2009, 2015, 2017), while the remaining selected records (*n* = 14) focused on adults.

*Measurement time points* ranged from 1-month pre-treatment (De Jongh et al., 1995) to 12 months follow-up (*n* = 9) (Öst et al., 1992, 1997a, 2001a,b; De Jongh et al., 1995; Öst, 1996; Götestam, 2002; Haukebo et al., 2008; Vika et al., 2009). Post-treatment assessment occurred immediately after the intervention (*n* = 9) (Öst et al., 1992; De Jongh et al., 1995; Öst, 1996; Haukebo et al., 2008; Vika et al., 2009; Muller et al., 2011; Moldovan and David, 2014; Hyett et al., 2018; Hemyari et al., 2019) or 1 week later (*n* = 8) (Öst et al., 1997a, 2001a,b; Götestam, 2002; Huey and Pan, 2006; Ollendick et al., 2009, 2015, 2017). The impact of the intervention was also evaluated after 2 weeks (*n* = 1) (Hyett et al., 2018), 1-month (*n* = 4) (De Jongh et al., 1995; Nuthall and Townend, 2006; Muller et al., 2011; Ollendick et al., 2015), 3 months (*n* = 1) (Nuthall and Townend, 2006), and 6 months (*n* = 4) (Götestam, 2002; Ollendick et al., 2009, 2015, 2017) across studies.

### The Impact of Single Session Therapy on Anxiety Disorders in Youth

All the studies involving youth (*n* = 4) implemented a 3-h CBT-exposure SST to reduce symptoms of specific phobias according to the Anxiety Disorders Interview Schedule for DSM-IV (ADIS)—child (C) and parent (P) versions. The sample size varied from 60 to 196 participants of both genders, aged 6–17 years across studies. Two studies compared the efficacy of two different forms of CBT-exposure (youth alone; SST vs. youth with a parent; A-SST) (Öst et al., 2001b; Ollendick et al., 2015), respectively, at 6 (Ollendick et al., 2015) and 12-month post-treatment (Öst et al., 2001b), and one of them also included a waiting list (WL) condition (Öst et al., 2001b). In both studies, anxiety levels decreased significantly over time (*p* < 0.001) with comparable trajectories across groups.

In the study by Ollendick et al. (2015), a trend (*p* = 0.07) toward a higher percentage of diagnosis-free participants was observed after 6 months in the SST group (67.39%), compared with the A-SST condition (49.02%), with older doing better than their younger counterpart. Also, a significantly higher parent self-efficacy was found in the A-SST group at all-time points, compared to caregivers of children in the SST group (*p* < 0.001).

Moreover, findings from the study by Öst et al. (2001b) showed that the SST group had a significantly better behavioral performance using the Behavioral Approach Tests (BAT) than both the parent-present group (*p* < 0.02) and the WL group (*p* < 0.0001), and the parent-present group also did better (*p* < 0.0001) than the WL condition. Despite no significant between-group differences were found for all the selected physiological and psychological measures, the treatment groups showed the most consistent changes, with maintained effects at follow-up.

Two studies, instead, examined the efficacy of a CBT-exposure SST compared with a single encounter of educational support therapy (EST) (Ollendick et al., 2009, 2017) at 1-week and 6 months follow-ups, and one of them also included a WL condition (Ollendick et al., 2009). Results from both studies showed that the two treatment conditions were effective in reducing anxiety symptoms over time (*p* < 0.001), more than the WL control condition (Ollendick et al., 2009). Moreover, in the study by Ollendick et al. (2009) SST resulted in an improved percentage of participants who were diagnosis free after 6 months (SST = 52% vs. EST = 21%), child ratings of anxiety during the behavioral avoidance test, and treatment satisfaction of the youth and their parents—regardless of whether the sample was American or Swedish. Also, a significant reduction of phobic beliefs measured with the phobic beliefs scale was observed over time points by Ollendick et al. (2017) in both conditions in favor of SST (*p* < 0.001), and it was found to predict lower anxiety levels.

### The Impact of Single Session Therapy on Anxiety Disorders in Adults

#### One Session Therapy vs. Multiple Sessions

Six studies compared the effect of one 3-h SST with four-sessions (Hemyari et al., 2019) or five-sessions (Öst et al., 1992, 1997a, 2001a; Haukebo et al., 2008; Vika et al., 2009) of CBT-exposure treatment (lasting between 5 and 8 h) in reducing symptoms of specific phobia in adults. Among these, one study also incorporated elements of cognitive restructuring to both conditions (Öst et al., 1997a). Moreover, two contributions (Öst et al., 2001a; Haukebo et al., 2008) included an additional WL control, of which Öst et al. (2001a) assigned the participants to four conditions: SST-CBT-exposure, five-sessions of CBT-exposure, five-sessions of CBT, or a WL.

The samples ranged from 28 to 55 participants of both genders aged 18–60 years across contributions, except for one study that enrolled only women (Hemyari et al., 2019). In four studies (Öst et al., 1992; Haukebo et al., 2008; Vika et al., 2009; Hemyari et al., 2019) participants were assessed before treatments delivery immediately after the interventions, while two studies tested the impact of the treatments after 1-week (Öst et al., 1997a, 2001a), and five records (Öst et al., 1992, 1997a, 2001a; Haukebo et al., 2008; Vika et al., 2009) had a further 12-month follow-up.

In all the selected studies a significantly greater anxiety symptoms improvement was observed over time in participants assigned to both the SST and multiple sessions groups, compared to the WL control.

Three studies (Öst et al., 1992, 1997a, 2001a) revealed a significant decrease in phobic symptoms measured by the Fear Survey Schedule III (FSS-III) up to 12-month follow-up (*p* < 0.0001) across conditions, but significantly greater improvements were observed in the treatment conditions, compared to WL (*p* < 0.05) (Öst et al., 2001a). General levels of anxiety were also evaluated, together with depressive symptoms, in three studies (Öst et al., 1992, 1997a, 2001a) using the Beck Anxiety Inventory and Beck Depression Inventory, respectively. Results showed significantly higher symptoms decreased (*p* < 0.0001) from baseline to 12-month follow-up in the intervention groups, compared to no-treatment (*p* < 0.05) (Öst et al., 2001a).

Moreover, two studies (Haukebo et al., 2008; Vika et al., 2009) specifically focused on reducing dental anxiety indicated a statistically significant decrease in the scores at the Dental Anxiety Scale over time across conditions (*p* < 0.001) (Haukebo et al., 2008; Vika et al., 2009). Both treatment conditions showed better outcomes than the WL control (*p* < 0.01) (Haukebo et al., 2008) but a significant reduction in phobic symptoms was observed only in the multi-session group at 12-month follow-up (*p* < 0.001) (Vika et al., 2009). Haukebo et al. (2008) also made use of the dental fear survey and noted a significant steady decrease in dental fear (*p* < 0.0001) in favor of the 5-CBT-E compared to SST (*p* < 0.05).

Both a planned single session and four-sessions of CBT-exposure treatment were also shown equally effective in reducing rat phobia through the Fear of Rats Questionnaire in one study (Hemyari et al., 2019).

Five out of six studies (Öst et al., 1992, 1997a, 2001a; Haukebo et al., 2008; Vika et al., 2009) also measured the behavioral response of the participants using the BAT.

From baseline to 12-month follow-up avoidance behaviors reduced significantly (*p* < 0.0001) with similar trajectories across treatment conditions in two studies (Öst et al., 1992; Vika et al., 2009), while Öst et al. (1997a,b) observed an initially significant decrease in anxiety symptoms (*p* < 0.0001) from baseline to 1-week follow-up, but a worsening after 1 year from treatment termination (*p* < 0.05) in both intervention groups, with inferior outcomes in the SST condition. Among those studies that also included a WL control (Öst et al., 2001a; Haukebo et al., 2008), participants in the treatment conditions also showed a significantly greater reduction in avoidance behaviors (*p* < 0.001) after 12 months from treatment termination. Furthermore, in the study by Öst et al. (2001a) participants in the SST had greater BAT-scores from pretreatment to 12-month follow-up than those assigned to the other treatment conditions (5-CBT-E and 5-CBT) (*p* < 0.0001).

In two studies (Öst et al., 1992, 2001a) physiological measures (heart rate, systolic, and diastolic pressure) confirmed the above self-report measure of anxiety symptoms fluctuations, as positive effects were registered from baseline to 12-month follow-up in both intervention groups (*p* < 0 < 0*.0*1).

#### One Session Therapy vs. One Session Therapy: Effects of Different SSTs

Four studies (Öst, 1996; Götestam, 2002; Huey and Pan, 2006; Muller et al., 2011) compared the effects of different SSTs for spider phobias. Sessions lasted between 27 min and 3 h.

Studies compared SST of CBT-exposure and modeling in small and large groups (Öst, 1996), a modeled version of CBT-exposure with a direct CBT-exposure and a video CBT-exposure conditions (Götestam, 2002), a culturally adapted CBT-exposure with standard CBT-exposure and a manualized self-help intervention (Huey and Pan, 2006), or a computer-based SST of CBT-exposure using a spider or neutral pictures (Muller et al., 2011). Moreover, two studies compared a 60-min SST of cognitive restructuring intervention with an information intervention for dental phobia (De Jongh et al., 1995), or 90-min imagery rescripting with verbal restructuring for social anxiety (Hyett et al., 2018) with WL controls. The sample ranged from 15 to 58 participants of both genders aged 18–65 years, except for two studies that enrolled only women (Öst, 1996; Muller et al., 2011).

Four studies (De Jongh et al., 1995; Öst, 1996; Muller et al., 2011; Hyett et al., 2018) made a pre-immediately post-intervention assessment, while two studies (Götestam, 2002; Huey and Pan, 2006) tested the intervention after 1-week. Follow-up periods were after 2 weeks (Hyett et al., 2018), 1 month (Muller et al., 2011), and 12-month (De Jongh et al., 1995; Öst, 1996; Götestam, 2002) from treatment termination.

Symptoms of phobia decreased significantly employing the FSS-III and the BAT measures from baseline to 12-month follow-up in both small and large groups—with more favorable results with fewer participants—and analogous heart rate, and blood pressure variability (*p* < 0.0001) (Öst, 1996).

In the treatment conditions (culturally adapted CBT-exposure vs. standard CBT-exposure) better FSS-III (*p* < 0.10), FTQ (Fearful Thoughts Questionnaire; *p* < 0.05), and BAT scores (*p* < 0.001) were also observed compared to the manualized self-help intervention control after 1 week from the end of the treatment (Huey and Pan, 2006).

The BAT (*p* < 0.001) and Fear of Spider Questionnaires (*p* = 0.004) further revealed that SST of computer-based self-help CBT treatment was more effective over a 1 month period when spider pictures were used, compared to neutral stimuli (Muller et al., 2011).

Negative beliefs contributing to symptoms of dental and spider phobias also reduced significantly at 1-month follow-up (*p* < 0.005) in the SST-cognitive restructuring group compared to the information and the WL interventions (De Jongh et al., 1995), and at 12-month follow-up (*p* = 0.001) among participants assigned to the direct exposure group compared to those in the modeled CBT-exposure and video CBT-exposure conditions (Götestam, 2002).

#### One Session Therapy vs. Waiting List Only

The impact of SST of CBT was compared with a WL condition only in two studies (Nuthall and Townend, 2006; Moldovan and David, 2014), aged 18–58 years. Findings revealed a significant reduction in symptoms of panic disorder among 36 respondents aged 18–58 years after 1 month from treatment termination in both the CBT (*p* = 0.004) and the WL groups (*p* = 0.026), but at 3-month follow-up, positive results were maintained only in the treatment condition (*p* = 0.01) (Nuthall and Townend, 2006). Moreover, one study applied 4–5 h of virtual reality-CBT on 32 adults suffering from social phobia, flight phobia, and acrophobia and revealed significant pre–post between-groups differences in the levels of distress, flight anxiety, and self-statement during public speaking (*p* < 0.05) (Moldovan and David, 2014) in favor of the treatment condition, while no meaningful variations in symptoms of general anxiety and acrophobia, and reduced rational and irrational beliefs were observed.

## Discussion

To our knowledge, this is the first study aimed at providing a summary of the available evidence on the efficacy of planned single-session psychological interventions in reducing anxiety symptoms in children, adolescence, and the adult population.

Collectively, the results from 18 RCTs support the benefits of a single therapeutic encounter in enhancing cognitive, behavioral, and physiological outcomes among people suffering from different anxiety disorders across treatment approaches, populations, and cultures, and allow tentative conclusions to be drawn about its effectiveness. Single-Session Therapy was found superior to no treatment in reducing anxiety symptoms, and it was also linked to changes in self-efficacy and overall treatment satisfaction in both children and adults.

These findings are not surprising in as much as the large majority of the selected studies incorporate single-session psychological interventions employing well-established cognitive-behavioral treatment procedures (i.e., participant modeling, reinforced practice, and systematic exposure).

Four RCTs examined the use of a single session of CBT-exposure treatment, compared to a dyadic condition (youth with parents), an educational support encounter, and/or a WL control among youth aged 6–17 years. Overall, significantly better outcomes were observed over time across groups, with the most consistent changes observed in the youth-alone group, compared with its parent-present counterpart.

These findings further support the relationship between treatment expectations and outcomes and highlight the importance for young people to take an active role over their problems to positively influence their beliefs about themselves, their ability to succeed, and possible solutions (Horne, 1999).

Indeed, SST operates under the assumption that the ongoing therapeutic encounter is the only available, and additional sessions may not be necessary. Therefore, part of the strength of this approach lies in its ability to empower patients to manage problems themselves (Cannistrà and Piccirilli, 2018)—with the consequent increase in their perceived ability to face a given challenge.

In practice, within a single therapeutic encounter, the professional seeks to positively influence the thoughts and behaviors of the individuals, while recognizing that many significant changes will occur outside the planned therapy process. This increases their control and responsibility for the problem and the magnitude of the treatment outcomes.

Single-session therapy might also reduce public and self-stigma related to mental illness, which can further weaken self-esteem and self-efficacy of the people, besides negatively affect the emotional well-being and personal relationships of the individuals (Livingston and Boyd, 2010) as well as harms help-seeking behavior, treatment adherence, and recovery (Gulliver et al., 2010).

The impact of a single session CBT-exposure intervention on anxiety disorders in adults was compared with the provision of multiple encounters of the same treatment in six studies (Öst et al., 1992, 1997a, 2001a; Haukebo et al., 2008; Vika et al., 2009; Hemyari et al., 2019), a different single session intervention in four records (Öst, 1996; Götestam, 2002; Huey and Pan, 2006; Muller et al., 2011), and a WL control only in two contributions (Nuthall and Townend, 2006; Moldovan and David, 2014). Findings revealed a significant anxiety reduction and—when measured—depressive symptoms (Öst et al., 1992, 1997a, 2001a) across time points, with a similar trajectory between one vs. multi-treatment-sessions, over and above the WL condition, except for two studies—in which a significant reduction in phobic symptoms was observed in favor of the multi-session group (Haukebo et al., 2008; Vika et al., 2009). Also, one contribution (Öst et al., 1997a) showed inferior outcomes at 12-month follow-up among participants receiving a single session treatment compared to their counterparts.

Despite the observation that SST did not show superior outcomes to multi-session treatments, these findings might partially support the cost-effectiveness of this approach.

Differences in symptom types and presentations can somewhat explain the absence of positive effects observed in a few studies. Some patients may require additional sessions, and or an alternative therapy as in the case where the person fails to respond adequately to first-line diagnosis and treatment. The shortest therapies might—therefore—be tried first and, if these do not lead to positive outcomes, longer-term approaches may be used.

Another possible reason is that re-exposure to triggers—without allowing time for habituation as happens over several sessions of exposure therapy—might have led to an increase in psychological distress.

In those studies comparing different one-session conditions more favorable long-term results were observed by employing a direct CBT-exposure treatment, compared with its modeled and video versions (Götestam, 2002), and with fewer participants when the intervention was provided in group format (Öst, 1996).

Moreover, cognitive restructuring therapy resulted superior to both information giving and no treatment (De Jongh et al., 1995), and a computer-based self-help CBT treatment was more effective when spider pictures were used, compared to neutral stimuli (Muller et al., 2011) after one month from treatment termination.

Better short-term results were also observed with a standard CBT-exposure intervention and its culturally adapted version, in contrast with a manualized self-help control (Huey and Pan, 2006), while no differences were found between imagery rescripting and verbal restructuring interventions, over and above the WL control (Hyett et al., 2018). Furthermore, both a single session of traditional CBTs (Nuthall and Townend, 2006) and virtual reality-CBT (Moldovan and David, 2014) produces superior outcomes than non-treatment.

### Limitations and Straights

While modified versions of the same treatment have been compared, a single session intervention was likened with multiple sessions of the same treatment, and comparisons have been made between a single session of a given therapeutic intervention and a no-treatment condition across the selected studies, no contribution likening different models of psychotherapy delivered in a single encounter for the treatment of anxiety disorders has been found and therefore discussed within the findings of the present contribution. This might be due to the fact that, despite a very extensive review covering established health and psychological databases has been implemented, gray literature searches have not been performed—thus possibly leading to the exclusion of important contributions.

Exclusion of relevant articles might also be attributable to the choice not to include in the present systematic review research on walk-in single-session—but only on planned SST interventions.

Still, walk-in services—by nature—do not provide further visits with the same professional, as people can ask for additional help but for a different problem. Furthermore, walk-in services do not allow a rigorous assessment of treatment outcomes for a specific disorder, as people can directly access psychological treatment without being placed on WLs or undergoing a diagnostic process.

Therefore, walk-in single-session and SST by appointment approaches differ in their characteristics, which must be carefully taken into account while conducting a review study on the topic to increase the reliability of research findings.

On the other hand, owing to the growing burden of anxiety disorders, the combined analysis of the impact that both SST approaches have in treating anxiety disorders might further enhance the efficacy of mental health services, making them even more accessible.

Therefore, future contributions should broaden the literature search, integrating the results of this first systematic review on the impact of planned SST on anxiety disorders with those coming from controlled studies on walk-in services for anxiety problems.

Moreover, methodological factors might have influenced the study findings, including the failure in several contributions to account for drop-outs rates that—together with the small sample sizes—might have decreased the likelihood to find real between-group differences for the selected outcomes in the short and long term. Further, it is possible that the interventions were not properly delivered or provided by inexperienced therapists—as studies did not attempt to ensure the quality of the intervention in any meaningful way.

Furthermore, the interventions varied widely in terms of therapy delivered, treatment format, and the use of digital tools among the selected studies, precluding speculation on the superiority of a specific experimental condition.

### Future Research and Practical Directions

Cognitive-behavioral therapy remains the first-line treatment for anxiety disorders in both youth and adults—as also noted by the National Institute for Health and Care Excellence's guidelines (Hofmann et al., 2012) and American Psychological Association (Hofmann et al., 2013)—as its theoretical models/mechanisms of change have been the most researched and are in line with the current mainstream paradigms of human mind and behavior (David et al., 2018). Further, concerning the comparability of remote and in-person treatment, findings from this review support those from previous research showing that common psychotherapies such as CBT can be just as effective on mood and anxiety disorders when delivered digitally (Stubbings et al., 2013; Arnedt et al., 2019; Bertuzzi et al., 2021; Probst et al., 2021). Given the expanded real-life options for exposure and novel needs of people, it is exciting to think about all the possible applications of CBT (i.e., within a single session encounter or remotely delivered). However, there is room for further improvement, as in many situations, there are patients who do not respond to CBT and/or relapse (Castelnuovo et al., 2011; Jackson et al., 2018). While many non-CBT psychotherapies have changed little in practice since their creation, CBT is an evolving form of therapy based on research. Therefore, more examinations on the application of less supported or controversial psychological treatments for anxiety disorders across the lifespan is required to validate their underlying constructs and mechanism of change. This should be paralleled by continuous improvements in CBT techniques to gradually move toward integrative scientific psychotherapy. Indeed, the findings of this systematic review support the adaptability and creativity of SST in reducing symptoms of anxiety. Since outcomes did not differ significantly with the combinations of several treatment components, a single session approach to therapy seems to impart benefit beyond the mere sum of its component interventions and appears to combine these elements uniquely.

Indeed, SST is meant to be a flexible approach, where many different techniques and methods can be applied. Testing its potential using various evidence-based approaches that adequately meet the needs of the patients within a given culture or context would contribute to a better understanding of the mechanisms of change in the field of mental health, besides strengthening the efficacy of mental health services.

In this contribution, SST has also been shown equally effective in diverse contexts and countries, but only the implementation of cross-cultural research can make the interpretations of findings more meaningful and would be able to further translate research evidence into clinical practice.

Furthermore—despite this study concluded that multi-session treatments are not superior to SST in reducing anxiety symptoms—none of the selected studies properly conducted cost and benefit analyses.

Whether clients who receive SST maintained their changes or continued to improve over the long term is also worth additional investigation. Therefore, future longitudinal research comprising longer follow-up periods and cost-benefit analysis is required to draw valid conclusions over the cost-effectiveness of a single therapeutic encounter in treating anxiety disorders (Castelnuovo et al., 2016).

Moreover, several important questions remain unanswered, namely, how moderators and mediators contribute to SST outcomes or the magnitude, variability, and generalizability of SST effects. Future directions for meta-analyses of SST outcome studies need to be considered in the presence of publication with fewer reporting biases that are likely to produce an appropriate quantitative summary.

## Conclusions

The findings reported here indicate that the SST is therapeutically effective in the treatment of youth and adults with anxiety problems. By providing a promptly delivered, client-centered assessment and intervention, SST has the potential to be highly cost-effective, to meet the need of most stakeholders, and to increase access to mental health services. Single-Session Therapy appears promising for both clinicians and researchers.

## Data Availability Statement

The original contributions presented in the study are included in the article/supplementary files, further inquiries can be directed to the corresponding author/s.

## Author Contributions

GP and FC: study conception and design. VB, GF, and CT: acquisition of data. GP, VB, GF, and CT: analysis and interpretation of data and drafting of the manuscript. FC, VG, EG, and GC: critical revision. All authors contributed to the article and approved the submitted version.

## Conflict of Interest

The authors declare that the research was conducted in the absence of any commercial or financial relationships that could be construed as a potential conflict of interest.

## Publisher's Note

All claims expressed in this article are solely those of the authors and do not necessarily represent those of their affiliated organizations, or those of the publisher, the editors and the reviewers. Any product that may be evaluated in this article, or claim that may be made by its manufacturer, is not guaranteed or endorsed by the publisher.
